# Corneal Alternations Induced by Topical Application of Benzalkonium Chloride in Rabbit

**DOI:** 10.1371/journal.pone.0026103

**Published:** 2011-10-12

**Authors:** Wensheng Chen, Zhiyuan Li, Jiaoyue Hu, Zhenhao Zhang, Lelei Chen, Yongxiong Chen, Zuguo Liu

**Affiliations:** Eye Institute and affiliated Xiamen Eye Center of Xiamen University, Fujian, China; University of Reading, United Kingdom

## Abstract

Benzalkonium chloride (BAC) is the most common preservative in ophthalmic preparations. Here, we investigated the corneal alternations in rabbits following exposure to BAC. Twenty-four adult male New Zealand albino rabbits were randomly divided into three groups. BAC at 0.01%, 0.05%, or 0.1% was applied twice daily to one eye each of rabbits for 4 days. The contralateral untreated eyes were used as control. Aqueous tear production and fluorescein staining scores of BAC-treated eyes were compared with those of controls. The structure of the central cornea was examined by in vivo confocal microscopy. Expression of mucin-5 subtype AC (MUC5AC) in conjunctiva was detected by immunostainig on cryosections. Corneal barrier function was assessed in terms of permeability to carboxy fluorescein (CF). The distribution and expression of ZO-1, a known marker of tight junction, and reorganization of the perijunctional actomyosin ring (PAMR) were examined by immunofluorescence analysis. Although there were no significant differences between control and BAC-treated eyes in Schirmer scores, corneal fluorescein scores and the number of conjunctival MUC5AC staining cells, in vivo confocal microscopy revealed significant epithelial and stromal defects in all BAC-treated corneas. Moreover, BAC at 0.1% resulted in significant increases in central corneal thickness and endothelial CF permeability, compared with those in control eyes, and endothelial cell damage with dislocation of ZO-1 and disruption of PAMR. Topical application of BAC can quickly impair the whole cornea without occurrence of dry eye. A high concentration of BAC breaks down the barrier integrity of corneal endothelium, concomitant with the disruption of PAMR and remodeling of apical junctional complex *in vivo*.

## Introduction

Preservatives are a major component of the ophthalmic preparations to provide antimicrobial activity and prevent decomposition of the active drug in multi-dose bottles. Benzalkonium chloride (BAC) is the most common preservative in ophthalmic preparations for its apparently good safety/efficacy profile [1]. Furthermore, as a soluble antimicrobial agent and cationic surfactant, BAC was found to have ability to enhance penetration of active compounds [2], [3]. However, a large number of experimental and clinical studies have shown that long-term use of topical drugs with BAC may induce ocular surface changes, causing ocular discomfort, tear film instability, loss of goblet cells, inflammation, conjunctival squamous metaplasia, epithelial apoptosis, subconjunctival fibrosis and the potential risk of failure for further glaucoma surgery [1].

BAC is most often used at a concentration of 0.01% (ranging from 0.004% to 0.02%) in ophthalmic preparations [4]. As long-term clinical testing with low concentration of BAC is difficult to conduct on animals [5], many researchers have investigated acute toxic effects of high concentrations of BAC (approximately 1-50 times the commercial concentrations) on the ocular surface. In 1944, Swan found that BAC causes punctuate disturbances to corneal epithelium at concentration of 0.04%, and edema and cellular desquamation with corneal lesions at a concentration of 0.1% [6]. Since then, much clinical and experimental evidence has been obtained to support the notion that the toxic effect of BAC on the ocular surface is primarily related to its concentration. Recently, Liang et al. showed that following short- and repeated exposure to 0.02% BAC, a large number of inflammatory cells were present in rabbit corneal epithelial basal layer and stroma [7]. Although a few studies have proven that topical application of high concentration of BAC (0.25% or 0.5%) induced corneal endothelial cell edema, even disappearance [5], [8], *in vivo* toxic effect of BAC on the barrier function of corneal endothelium remains unclear.

Aqueous humor movements into the cornea are required as a source of nutrition for cells residing within the corneal stroma. The corneal stroma has a tendency to swell due to the presence of non-diffusible, negatively charged molecules such as hydrophilic glycosaminoglycans. The corneal endothelium is thought to be solely responsible for the maintenance of stromal deturgescence, which is prerequisite for corneal transparency [9], [10]. In healthy cornea, the endothelium forms a semi-permeable barrier that regulates fluid and solute exchange between the nutrient-rich aqueous humor and avascular corneal stroma, and tight junctions are an integral component of this barrier [10]–[12]. The tight junctions of the corneal endothelium are focally present around endothelial cells and serve to prevent the movement of aqueous humor from anterior chamber into corneal stroma. The tight junction complex in the corneal endothelium includes transmembrane proteins such as claudin and occludin; membrane-associated proteins such as zonula occludens (ZO)-1; and actin filaments [11]. ZO-1 plays an important role in maintaining the barrier function and has been considered a maker of the tight junction in the corneal endothelium [11], [13]. The corneal endothelium, similar to the epithelium, exhibits a thick band of actin cytoskeleton at the apical junctional complex (AJC), which has been referred to as the peri-junctional actomyosin ring (PAMR). This pool of actin cytoskeleton is structurally and functionally coupled to cytoplasmic domains of tight junctions and adherens junctions through linker protein such as ZO-1. It has been demonstrated that PAMR disruption is implicated in loss of the barrier integrity in the corneal endothelium [14], [15].

In this study, we evaluated the corneal alternations induced by topical application of BAC in rabbits. We were particularly interested in investigating the *in vivo* toxic effect of BAC on the barrier function of the corneal endothelium.

## Methods

### Animals and BAC Treatment

A total of 24 male white New Zealand rabbits (obtained from Shanghai Medical Laboratory Animal Center, Shanghai, China) weighing between 2 and 2.5 kg were randomly assigned to three groups of 8 rabbits each. The animals were housed individually in cages at constant room temperature (19–23°C) and humidity of 40%–50% with a constant 12-hour light-dark cycle. They were fed with chow and water ad libitum. Different concentration of BAC- 0.01%, 0.05%, or 0.1% (Sigma, St. Louis, MO) was applied to one eye of each rabbit twice daily (8 AM and 5 PM) for 4 days, with the second eye of each animal serving as a BAC-free control. All the experimental and animal care procedures were performed in compliance with the ARVO Statement for the Use of Animal in Ophthalmic and Vision Research and approved by the animal ethics committee of Xiamen University School of Medicine (approval ID: XMUMC 2009-01-18).

### Aqueous Tear Production

Rabbits were injected intraperitoneally with a mixture of xylazine (1 mg/kg body weight; Bayer, Shawnee Mission, KS) and sodium pentobarbital (20 mg/kg; Abbott Laboratories, North Chicago, IL) to keep the animals immobile. Five minutes after topical application of proparacaine (Alcaine; Alcon, Fort Worth, TX), tear production was measured with Schirmer paper strip (Tianjin Jingming New Technology Development Co., Ltd., Tianjin, China). The Schirmer paper strip was inserted into the lower conjunctival fornix and left in place for 5 minutes. After the strip was removed, the amount of wetting in millimeter was recorded to an accuracy of 0.5 mm.

### Slit-lamp Evaluation and Fluorescein Test

The ocular surface was evaluated using a slit-lamp biomicroscope (BQ900® Haag-Streit, Bern, Switzerland). Two microliters of 1% sodium fluorescein was dropped into the conjunctival sac, and the excess fluorescein was washed out with saline. Cornea was examined and graded under the slit-lamp biomicroscope with a cobalt blue filter [16].

### In Vivo Confocal Microscopy

After fluorescein staining analysis, a laser scanning confocal microscopy (Heidelberg Retina Tomography (HRTIII)/Rostock Corneal Module [RCM]; Heidelberg Engineering GmbH, Dossenhein, Germany) was used to examine corneas in vivo. The use of this microscope has been described previously [17]. A drop of carbomer gel (Alcon Laboratories, Fort Worth, TX) was applied as coupling medium between the applanating lens and the cornea. A diode laser is used as a light source with a wavelength of 670-nm. The objective of the microscope is immersion lens (Olympus, Hamburg, Germany), magnification × 60, which is covered by a polymethyl methacrylate cap. Images consist of 384 × 384 pixels, allowing a scanning area of 400 µm^2^ with lateral and vertical resolutions of both 1 µm and a magnification up to 800 times. The center of the cap was applanated onto the central cornea by adjusting the controller, and in vivo digital images of the cornea were visualized directly on the computer screen. The central cornea was examined and more than 10 images were taken for each of the following structure: superficial and basal epithelium, stroma, and endothelium. The mean central corneal thickness was calculated based on the depth difference between the most superficial epithelium and the endothelium and was recorded as the average of a minimum of three individual acquisitions. All measurements were performed by a single investigator masked to the specific experimental conditions. At the end, the epithelial cell size, epithelial basal cell and endothelial cell density were automatically calculated using the tomography-associated software. Cell density was recorded as cells per square millimeter. Based on 10 images, the means and standard deviations were calculated for each parameter.

### Measurement of Permeability to Carboxy Fluorescein (CF)

Corneal epithelial barrier function was evaluated on the basis of measurement of corneal permeability to CF (0.3%, Cohasset, MA). The fluorophotometric methods were modified for rabbits from a previous report in rat [18]. In brief, 10 minutes after 40 µL of CF was applied to each ocular surface, the animals (n = 3 per group) were euthanized with overdose of pentobarbital sodium and cornea were excised. Each cornea was washed 3 times in 1mL of balanced salt solution (BBS; Alcon Laboratories, Fort Worth, TX) for 5 minutes per wash, and places in a tube with a tube with 1mL BBS. Each tube was wrapped in aluminum foil to protect the solution from light and placed on an orbital shaker for 90 minutes. The concentration of CF (nmol/µL) was measured by using a Gilfrod Fluoro IV fluormeter (Corning, Oberlin, OH).

Under a surgical microscopy, aqueous humor (0.1ml) was removed from the anterior chamber with 33G NanoFil Injectior System (World Precision Instruments, Sarasota, FL, USA) through the lateral corneal limbus. Twenty minutes after 0.1 µL of CF was injected into the anterior chamber, the animals (n = 3 per group) were euthanized. The corneas were excised, and endothelial permeability to CF was measured as described above.

### Immunofluorescein Staining

After in vivo examinations, rabbits (n = 5 per group) were euthanized after intravenous injection of a lethal dose of pentobarbital sodium. The eyes (including bulbar conjunctiva) were enucleated, and the nasal and temporal bulbar conjunctivas were snap-frozen in liquid nitrogen and cut into 8-µm -thick sections. Sections were fixed in acetone for 10 minutes at 4°C. After washing in phosphate-buffer saline (PBS), the sections were incubated with 1% bovine serum albumin (BSA) to block nonspecific binding, and then overlaid with a 1∶50 dilution of mouse anti-rabbit MUC5AC antibody (Abcam, Cambridge, UK) overnight at 4°C. The second day, the sections were washed with PBS, incubated with a 1∶100 dilution of fluorescein isothiocyanate (FICT)-conjugated goat anti-mouse IgG (Cell Signaling Technology, Inc., Danvers, MA) for 1 hour at 4°C, followed by three washed in PBS and nuclei counterstaining with 1∶100 dilution of Hoechst 33342 dye (Sigma).

Under a dissecting microscope (Model SZ40; Olympus, Tokyo, Japan), the retina, lens, and iris were discarded and four incisions were made in each cornea. Each cornea was fixed in situ in PBS with 4% paraformaldehyde for 5 minutes, and then was permeabilized with acetone for 3 minutes at -20°C. Subsequently, the corneas were washed in PBS with 1% Triton X-100 and 1% dimethyl sulfoxide (DMSO; TD buffer) as described previously [19], [20]. To block nonspecific staining, tissue blocks were incubated in 1% BSA diluted in TD buffer for 1 hour. Then, the tissues were placed in 1∶100 of mouse anti-rabbit ZO-1 antibody (Zymed, Carlsbad, CA) for 8 hours with agitation at 4°C. Next, the tissues were washed with TD buffer, placed in Alexa Fluor 488-conjugated secondary antibody (1∶500 dilution; Molecular Probes; Eugene, OR) for 1 hour at 4°C. After distilled water wash, the whole-mount cornea tissues were mounted endothelial side up on a slide and stained with a nuclear fluorescence dye, 4,6-diamidino-2-phenylindole (DAPI, Vector, Laboratories, Burlingame CA).

For PAMR staining, the whole mount corneas were incubated in 1∶1000 of Texas-red conjugated phalloidin (Molecular Prbes, Eugene, OR) overnight with agitation for 4 hours at 4°C. The tissues were rinsed three times in PBS (5 minutes per rinse), and then the whole mount corneas were mount epithelial or endothelial side up on a slide and stained with DAPI. The tissues and sections were all examined with a laser confocal microscopy (Olympus Fluoview 1000; Olympus, Japan).

### Statistical Analysis

Quantitative data are presented as mean ± SD and were analyzed by Dunnett multiple comparison test. P<0.05 was considered statistically significant.

## Results

### Aqueous Tear Production and Fluorescence Staining

**Figure 1:**
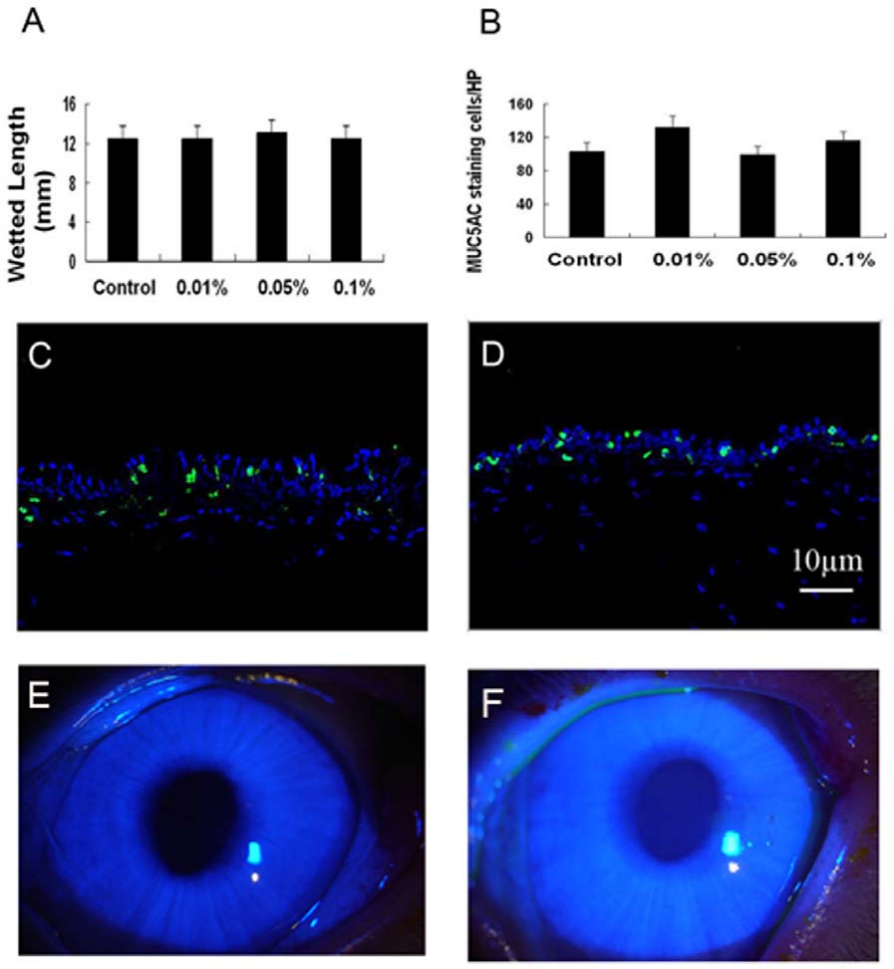
Toxic effects of BAC on aqueous tear production and conjunctival MUC5AC staining cell density. (A)Schirmer test. (B) conjunctival MUC5AC staining cell density. Representative images of immunofluorescence staining on conjunctival cryosections showing MUC5AC staining (*green*) with DAPI as nuclear counterstaining *(blue*). (C) Control group. (D) 0.1% BAC treated group. Representative images of corneal fluorescein staining showing no substantial fluorescein staining was apparent in both control (E) and 0.1% BAC treated (F) eyes. There were no statistically significant differences between the BAC-treated and the control groups in aqueous tear production and conjunctival MUC5AC staning cell density 4 days after BAC treatment (Dunnett test). Data show mean ± SD of values from 4 eyes per group.

There was no statistically difference between the BAC-treated and control eyes in aqueous tear production (P>0.1) (Fig. 1A). In addition, no substantial fluorescein staining was detected in the eyes treated with BAC, even at the highest concentration (Fig.1F).

### *In Vivo* Confocal Microscopy Analysis

**Figure 2:**
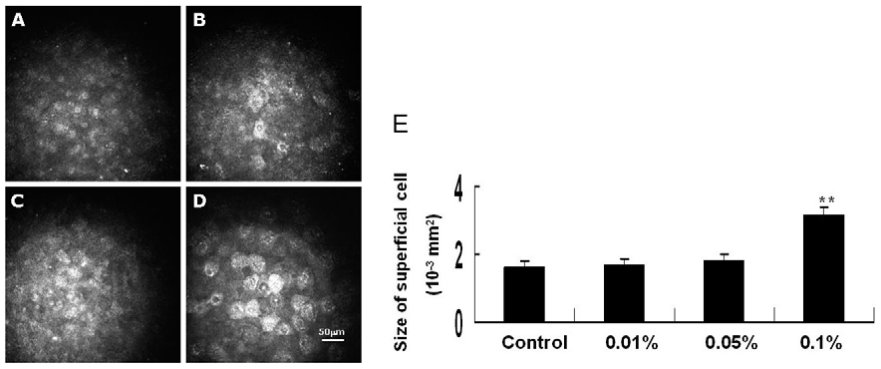
Toxic effect of BAC on the morphology of rabbit corneal epithelial superficial cells. Representative in vivo confocal images of the corneal epithelium in different groups. (A) Untreated control. (B) 0.01% BAC. (C) 0.05% BAC. (D) 0.1% BAC. Mean cell size at the epithelial surface was shown in (E). Note that the size of surface cells in the corneal epithelium of eyes treated with 0.1% BAC was significantly larger than that of control eyes. Data are mean ± SD of values from eight eyes per group. ** P<0.01 (Dunnett test).

The corneal superficial epithelial cells of control rabbits had a polygonal mosaic appearance with brightly reflective nuclei (Fig. 2A). All of the BAC-treated eyes displayed various abnormalities of the corneal superficial epithelial cells, including partial desquamation of epithelial cells, irregular cells shapes, anisocytosis and loss cell borders, abnormal reflectivity patterns, swollen (Fig.2B-D). Superficial epithelial cells were clearly observed in the eyes treated with 0.1% BAC, and the size of these cells was significantly larger, by 93.7%, than that of control eyes (Fig. 2E). These larger cells had a brightly reflective round mosaic appearance with dark nuclei (Fig. 2D).

**Figure 3:**
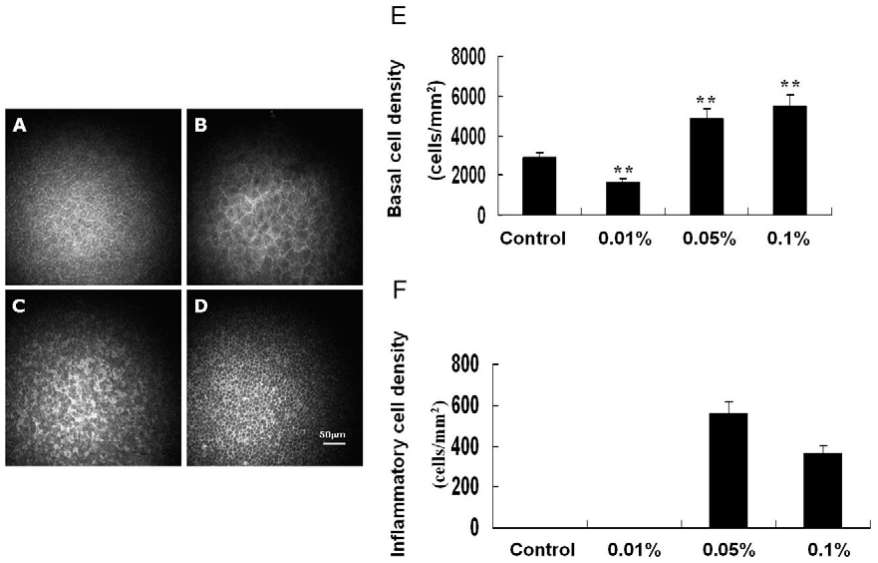
Toxic effects of BAC on the morphology of rabbit corneal epithelial basal layer. Representative in vivo confocal images of the corneal epithelium in different groups. (A) Untreated control. (B) 0.01% BAC. (C) 0.05% BAC. (D) 0.1% BAC. Mean basal cell and inflammatory cell densities were shown in (E) and (F), respectively. Data are mean ± SD of values from eight eyes per group. ** P<0.01 (Dunnett test).

The control basal epithelial cells appear as regular mosaic of dark cell bodies with light, narrow inter-cellular borders (Fig. 3A). The 0.01% BAC treatment did not induce any inflammatory cell infiltration in the epithelial basal layer, but the density of basal cells was significantly decreased, compared with those of normal and higher BAC concentration eyes (Fig. 3B, 3E). The 0.05% and 0.1% BAC induced inflammatory cell infiltration in the basal epithelium. The inflammatory cells were counted, giving densities of 560.3±28.3 and 368.2±16.5 cells/mm^2^ in the eyes treated with 0.05% BAC and 0.1% BAC, respectively. At both of the concentrations, the number of basal cells was significantly higher than that of control (Fig. 3C-E).

**Figure 4:**
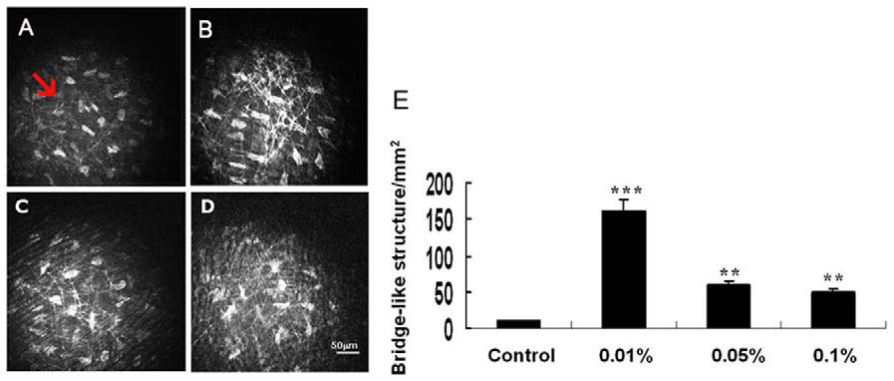
Toxic effects of BAC on the morphology of rabbit corneal anterior stroma. Representative in vivo confocal images of corneal anterior stroma in different groups. (A) Untreated control. (B) 0.01% BAC. (C) 0.05% BAC. (D) 0.1% BAC. Mean membrane bridge-like structure (A, arrow) was shown in (E). Note the dramatic difference in membrane bridge-like structure between control and BAC-treated eyes. Data are mean ± SD of values from eight eyes per group. ** P<0.01, *** P<0.001 (Dunnett test).

In control anterior stroma, keratocyte nuclei were seen as bright objects against a dark background. Hyperreflective membrane bridge-like structures (<0.5 µm diameter) were occasionally observed, and some of these structures formed distinct intercellular bridge between two or more cells (Fig 4A, arrow). These highly distinctive intercellular membrane bridge-like structures were generally short and straight and largely resemble membrane nanotubes observed in the mouse cornea.^21^ The abundance of these structures was significantly increased from 10.1±1.1 per square millimeter in the control to 160±15.8 per square millimeter in the corneas treated with 0.01% BAC, suggesting that they play an important role *in vivo* in cell-cell communication during the BAC-induced inflammation (Fig. 4B, 4E). The 0.05% and 0.1% BAC induced whole stromal disorganization, with loss of normal cell borders. The numbers of membrane bridge-like structures in both groups were decreased compared with 0.01% BAC treated rabbits, and yet still significantly higher than in the control (Fig. 4C-E).

**Figure 5:**
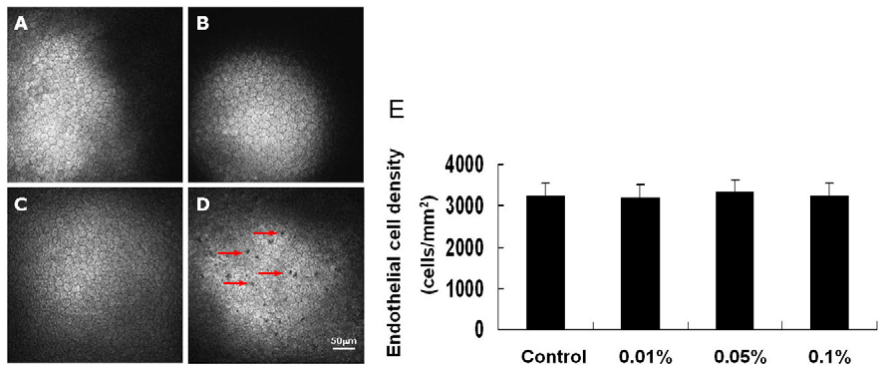
Toxic effects of BAC on the morphology of the rabbit corneal endothelium. Representative in vivo confocal microscopic images of the corneal endothelium in different groups. (A) Untreated control. (B) 0.01% BAC. (C) 0.05% BAC. (D) 0.1% BAC. There was no significant difference between BAC at lower concentrations treated and control eyes in the endothelial morphology. However, in eyes treated with 0.1% BAC, a number of hyporeflective spots were present at endothelial cell-cell borders (D, arrow). The mean endothelial cell density did not reach statistically significant levels in eyes treated with BAC when compared with control eyes (E). Data are mean ± SD of values from eight eyes per group.

The control endothelial cells had a polygonal mosaic appearance, with some larger hexagonal cells also apparent (Fig. 5A). Application of 0.01% and 0.05% BAC seemed not to inflict significant damage on the endothelial cells (Fig.5B, 5C). However, 0.1% BAC treated rabbits exhibited significant abnormal endothelial change, with presence of hyporeflective spots at many cell-cell borders (Fig. 5D, arrow). Cell density in the endothelium was not significantly affected by BAC treatment (Fig. 5E).

**Figure 6:**
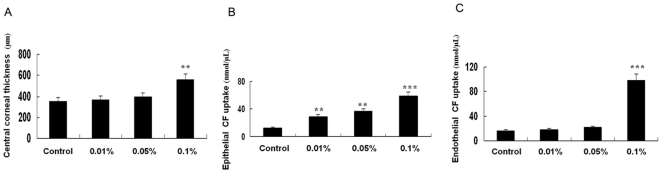
Toxic effects of BAC on central corneal thickness and CF uptake in the rabbit. (A) Central corneal thickness. (B) Epithelial CF uptake. (C) Endothelial CF uptake. Note that epithelial CF uptake was significantly increased in all BAC treated corneas, whereas central corneal thickness and endothelial CF uptake were only significantly increased in eyes treated with 0.1% BAC compared with those in control eyes. Data are mean ± SD of values from eight eyes per group. ** P<0.01, *** P<0.01 (Dunnett test).

We also measured central corneal thickness in the BAC-treated and control eyes. In control eyes, the mean central cornea thickness was 355±6 µm. Central corneal thicknesses was significantly increased by 57.2% in eyes that received 0.1% BAC, but BAC at lower concentration did not induce significant change in mean corneal thickness (Fig. 6A). These data thus suggested that higher BAC concentration leads to corneal edema as a result of a loss of corneal endothelial barrier function.

### Permeability Measurement

As shown in Figure 6B, treatment with BAC significantly increases epithelial permeability, compared with untreated control. Endothelial permeabilities measured for control versus 0.1% BAC treated corneas equaled 16 ± 5.2 nmol/µL and 125 ± 14.7 nmol/µL. These values were significantly different (*P*<0.001). In contrast, there was no significant difference of endothelial CF uptake between control and lower BAC concentration treated corneas (Fig. 6C).

### Immunofluorescence Staining Analysis

Immunofluorescence staining of conjunctival tissues with MUC5AC antibody revealed that MUC5AC positive cells were located in the nasal and temporal bulbar conjunctivas of BAC-treated and control eyes (Fig. 1C, 1D). Statistical analysis showed no significant difference between the BAC-treated and control eyes in the density of conjunctival MUC5AC positive cells (Fig. 1B).

**Figure 7:**
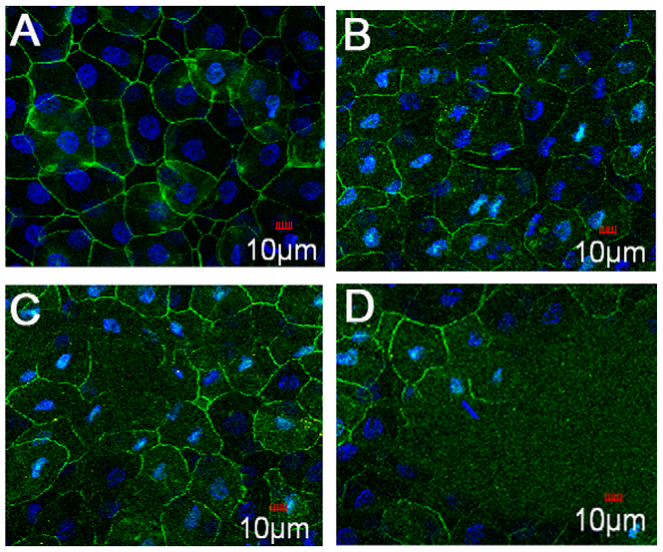
Toxic effects of BAC on localization of ZO-1 in the rabbit corneal epithelium. Corneal tissue blocks prepared from a control eye (A) or from eyes treated with 0.01% (B), 0.05% (C) or 0.1% BAC (D). ZO-1 staining was observed as a continuous linear pattern along with superficial cell-cell borders in normal rabbit corneal epithelial cells. In contrast, ZO-1 staining was patchy and discontinuous in eyes treated with BAC (B, C, and D). Note that BAC induced disappearance of ZO-1 in a concentration-dependent manner.

We next examined the effect of BAC on the expression of ZO-1 in the corneal epithelium. In the control corneas, ZO-1 is localized contiguously at the superficial cell-cell boundaries and accordingly stained uniformly at the cell borders (Fig. 7A). In contrast, ZO-1 staining was patchy and discontinuous in eyes treated with BAC (Fig. 7B-D). These observations thus suggested that BAC induced disruption of the localization of ZO-1 and loss of superficial cells in many areas of the epithelium.

**Figure 8:**
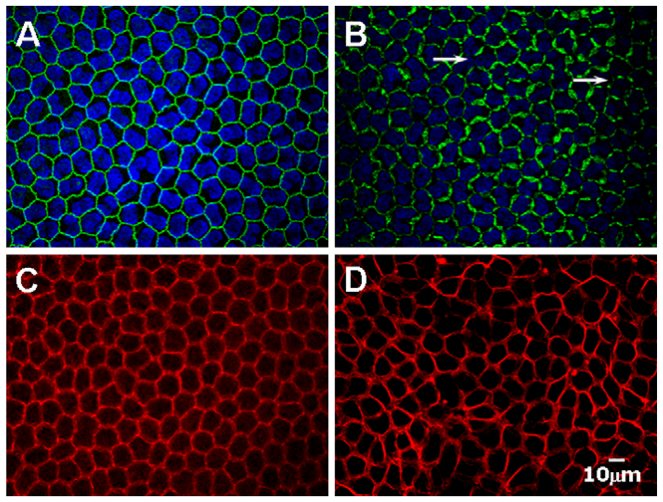
Toxic effects of BAC on the PAMR and distribution of ZO-1 in the corneal endothelium. (A, C) Untreated control eyes. (B, D) 0.1% BAC treated eyes. In untreated eyes, ZO-1 distribution is contiguous at the endothelial cell-cell border (A) and the characteristic organization of cortical actin with intact PAMP is observed (C). In contrast, BAC at 0.1% induced the dispersion of ZO-1(B), as evidence by discontinuities in ZO-1 distribution (C, arrow) and the disruption of PAMR (D).

To further corroborate presence of significant damage on the endothelial cell-cell borders, as revealed by in vivo confocal microscopy, we next examined the organization of the AJC. As shown by confocal images in Figure 8, a continuous linear pattern of ZO-1 staining at the boundaries of adjacent endothelial cells was found in the control eyes (Fig. 8). In both the 0.01% and 0.05% BAC-treated eyes, the pattern of ZO-1 distribution is similar to that of control (data not shown). A contiguous distribution of ZO-1 at intercellular border was found to be retracted and dislocation from the cell-cell border in eyes treated by 0.1% BAC, as evidenced by discontinuities in ZO-1 localization (Fig. 8B, arrow). These observations thus suggested that high BAC concentration could disrupt the TJs in the rabbit corneal endothelium in vivo.

We also investigated remodeling of the PAMR in response to BAC in vivo. PAMR is formed by the actin cytoskeleton at the AJC, and is structurally and functionally coupled to cytoplasmic domains of TJs and AJs.^11^ In control and BAC at low concentration treated eyes; F-actin formed a characteristic dense bend of PAMR (Fig 8C). In contrast, PAMR compacted into a contractile ring in 0.1% BAC treated eyes (Figure. 8D).

## Discussion

BAC is the most frequently used preservative in multi-dose eyes drops for its apparently good safety/efficacy profile. However, the generally good bactericidal outcomes have tended to overshadow the significant number of patients who undergo ocular discomfort after long-term use. Therefore, prophylaxis and treatment of toxic reactions associated with BAC have become important clinical issues. In the present study, we utilized an animal model to examine the toxic effect of BAC on the whole cornea. Our choice of different concentrations of BAC in this experiment was based on clinical practice and previous experimental studies. BAC is most often used in ophthalmic preparations at a concentration of 0.01% [4]. BAC at 0.1% was found to cause corneal edema [6] and was used to develop a rabbit dry eye model [21]. The 0.05% BAC concentration was added in this set of experiments, so as to better characterize the changes in the cornea found between the 0.01% and the 0.1% BAC concentrations.

We have now shown that topical application of BAC, even at a low concentration, breaks down rabbit corneal epithelial barrier function, as revealed by measurement of permeability to CF. ZO-1, which is a marker of tight junction of corneal epithelium, is contiguous at superficial cell-cell border [22], [23], and its dislocation is an index of the loss of tight junction integrity [24], [25]. In this study, we found that in control eyes, ZO-1 distribution was contiguous at the cell-cell borders at the surface of the rabbit corneal epithelium. In contrast, in BAC treated corneas, ZO-1 immunoreactivity was patchy and discontinuous in the superficial epithelium. Recent studies have proven that exposure to BAC induce a continuous decline in corneal transepithelial electric resistance in rabbit [26], [27]. Our study extends these finding and confirms that topical application of BAC disrupt the tight junctions between superficial cells in the rabbit corneal epithelium in vivo. We found that the highest BAC concentration used here (0.1%) not only resulted in a significant increase in the size of cells at the surface of the corneal epithelium, but also induced maximum corneal surface barrier damage, as shown by the severe CF uptake. These findings suggest that BAC at high concentration causes corneal surface epithelial cell edema and more adversely affect the barrier integrity of the corneal epithelium.

Our results showed a dose-response relationship for BAC-treated corneal epithelial basal layer, with low (0.01%) BAC concentration inducing basal cell edema, whereas the high (0.05% and 0.1%) BAC concentrations caused inflammation and increase in cell density. Liang et al reported that topical repeated application of BAC induced important inflammatory cell infiltration in rabbit corneal epithelial basal layer and stroma [7]. In 3D-reconstituted corneal epithelium, Pauly et al found that the number of proliferative cells increased after BAC treatment [28]. Our results were consistent with these studies, showing that topical application of BAC quickly impairs the corneal epithelium.

Membrane nanotubes that connect two or more cells have been found *in vitro* in a number of cells types including rat pheochromocytoma cell lines, normal rat kidney cells [29], and primary cultures of dendritic cells (DCs), macrophages, human peripheral blood NK cells, and B cells [30]. Recently, long and fine (<0.8 µm in diameter) membrane nanotube-like structures on bone marrow-derived major histocompatibility (MHC) class II+ DCs have been documented in the corneal stroma of normal mouse. The frequency of these nanotubes was significantly increased in corneas subjected to trauma and LPS, suggesting that these structures play an important role in vivo in cell-cell communication between widely spaced dendritic cells during inflammation [31]. Using *in vivo* confocal microscopy, we found that a few of long and hypereflective membrane bridge-like structures (<0.5 µm in diameter) were distributed in normal corneal anterior stroma. Four days after application of 0.01% BAC, the number of membrane bridge-like structures in anterior central stroma increased from 10.1±1.1 per square millimeter to 160±15.8 per square millimeter. Such a rapid and significant modulation of membrane bridge-like structures density suggests that they serve as critical structures in BAC at low concentration inducing corneal pathological changes. We found that the density of these structures was less increased in the eyes treated with 0.05 % and 0.1% BAC, probably because high concentration primarily caused deep tissue damage. To our knowledge, we are the first group to report the existence of the long and hypereflective membrane bridge-like structures in the central corneal stroma of the rabbit eye. Further studies are needed to determine whether the structures were membrane nanotubes that express MHC class II antigen.

Xiong et al. have reported significant decreases in Schirmer score and goblet cell density and increase in fluorescein scores in rabbits after 7 days treatment of 0.1% BAC twice daily [21]. They suggested that topical application of 0.1% BAC can induce a rabbit dry eye model, which is suitable for studying human dry eye syndrome. In the present study, we have found significant whole corneal defect without significant changes in aqueous tear production and corneal fluorescein scores after exposure to 0.1% BAC for 4 days. We noted that there was no significant difference of the number of MUC5AC cells between BAC-treated and control eyes. We also noted that even lower concentration of BAC could induce significant corneal stromal alterations. Our results, taken together, indicate that the rabbit cornea is very sensitive to the toxicity of BAC. Therefore, the direct toxic effect on the whole cornea should be taken into account if BAC is used to establish an animal dry eye model.

Pauly et al. performed a series of toxicological assessments in the rat eye after instillations of BAC [8]. They found that BAC at low concentrations (0.01% and 0.1%) induced damage restricted to the the epithelium, whereas the highest concentrations (0.25% and 0.5%) caused epithelial denudation as well as major stromal and endothelial damage. In our study, in vivo confocal microscopy revealed destructuring of the corneal stroma with presence of a lot of membrane bridge-like structures in eyes treated by lower (0.01% and 0.05%) concentration of BAC. Moreover, 0.1% BAC induced significant corneal endothelial cell defects. The reason for these discrepancies is not immediately clear; one possible explanation is due to species differences.

An important function of the corneal endothelium is to provide a barrier to the aqueous humor movements. The tight junction plays an important role in the establishment and maintenance of the barrier function of the endothelium [32], [33]. The tight junctions of corneal endothelium can be disrupted as a result of aging, inflammatory reaction, iatrogenic injury, and genetic disorders such as Fuch's dystrophy [34]. Our data suggest that topical application of high BAC concentration can induce loss of barrier integrity in the rabbit corneal endothelium *in vivo*. There are several lines of evidence to support this notion: First, application of 0.1% BAC, but not that of lower BAC concentrations, resulted in significant increases in central corneal thickness and endothelial permeability, compared with those in control eyes (P<0.01). This finding means that high BAC concentration can lead to significant corneal edema as a result of a loss of corneal barrier function. Second, in vivo confocal microscopy revealed presence of hyporeflective spots at many endothelial cell-cell borders in 0.1% BAC treated eyes, indicating that high BAC concentration inflict significant damage on the endothelial cell junctions. Third, in control and low concentrations of BAC-treated eyes, ZO-1 expression exhibited a continuous linear pattern of at cell-cell boundaries in the endothelium, whereas in eyes treat with 0.1% BAC, ZO-1 immunoreactivity was discontinuous, indicating the disruption of tight junctions. Moreover, 0.1% BAC induced the compaction of PAMR into a contractile ring. Taken together, the present study clearly shows that topical application of high concentration of BAC can not only damage the corneal epithelium and stroma, but also break down the barrier integrity in the rabbit corneal endothelium *in vivo*.

In summary, our study demonstrated that topical application of BAC can quickly impair the whole cornea without occurrence of dry eye. BAC at high concentration induces disruption of the barrier integrity in the corneal endothelium. These findings may provide additional information for our understanding of the toxic effect of BAC on the cornea.
